# The genome sequence of the Northern Summer Mayfly, *Siphlonurus alternatus *(Say, 1824)

**DOI:** 10.12688/wellcomeopenres.20172.1

**Published:** 2023-10-23

**Authors:** Andrew Farr, Craig R. Macadam, Natural History Museum Genome Acquisition Lab, Darwin Tree of Life Barcoding collective, Wellcome Sanger Institute Tree of Life programme, Wellcome Sanger Institute Scientific Operations: DNA Pipelines collective, Tree of Life Core Informatics collective, Darwin Tree of Life Consortium

**Affiliations:** 1 Independent researcher, Hailsham, England, UK; 2 Buglife – The Invertebrate Conservation Trust, Stirling, Scotland, UK

**Keywords:** Siphlonurus alternatus, Northern Summer Mayfly, genome sequence, chromosomal, Ephemeroptera

## Abstract

We present a genome assembly from an individual male *Siphlonurus alternatus* (the Northern Summer Mayfly; Arthropoda; Insecta; Ephemeroptera; Siphlonuridae). The genome sequence is 455.8 megabases in span. Most of the assembly is scaffolded into 11 chromosomal pseudomolecules, including the X sex chromosome. The mitochondrial genome has also been assembled and is 19.36 kilobases in length.

## Species taxonomy

Eukaryota; Metazoa; Eumetazoa; Bilateria; Protostomia; Ecdysozoa; Panarthropoda; Arthropoda; Mandibulata; Pancrustacea; Hexapoda; Insecta; Dicondylia; Pterygota; Palaeoptera; Ephemeroptera; Pisciforma; Siphlonuridae; *Siphlonurus*; *Siphlonurus alternatus* (Say, 1824) (NCBI:txid248243).

## Background

**Figure 1.**
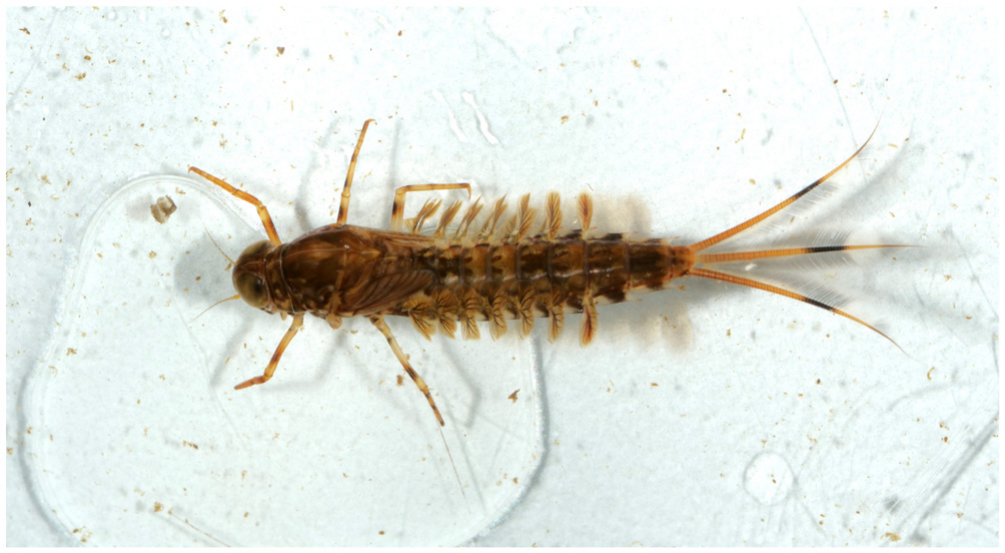
Photograph of the *Siphlonurus alternatus* specimen used for RNA sequencing.

*Siphlonurus alternatus* ( Figure 1) is a Holarctic species found in Canada and the northern United States, and across Northern Europe, particularly Fennoscandia ( GBIF Secretariat, 2023). In Britain and Ireland, it is a northern species with a highly localised distribution. It is anticipated that future surveys in south-west Scotland will turn up further records of this species ( Macadam, 2019).

This species is likely to be a eurytherm and is typically found in the middle reaches of watercourses ( Buffagni *et al.*, 2009). It is found at a range of altitudes, from the foothills to lowland watercourses. Larvae of this species typically live in deep pools in rivers and streams, but can also be found in calcareous lakes ( Bratton, 1990; Kimmins, 1932). The large nymphs are good swimmers and typically swim in short, darting bursts ( Elliott *et al.*, 1988).

*Siphlonurus alternatus* is univoltine, overwintering as eggs and emerging as adults between May and August ( Elliott *et al.*, 1988; Landa, 1968). Emergence of the adults typically takes place during daylight hours ( Hirvenoja, 1964), and males of this species can be found swarming at dawn and dusk over light patches of substrate on the bed of the water body or floating plants such as water-lilies ( Savolainen, 1978). The larvae feed by gathering or collecting fine particulate organic detritus from the sediment ( Elliott *et al.*, 1988).

The genome sequence for *Siphlonurus alternatus* will aid in understanding the biology, physiology and ecology of the species.

## Genome sequence report

The genome was sequenced from one male *Siphlonurus alternatus* collected from Drumpail Burn, Scotland (54.92, –4.77). A total of 53-fold coverage in Pacific Biosciences single-molecule HiFi long reads was generated. Primary assembly contigs were scaffolded with chromosome conformation Hi-C data. Manual assembly curation corrected 31 missing joins or mis-joins and removed 10 haplotypic duplications, reducing the assembly length by 0.69% and the scaffold number by 12.9%, and increasing the scaffold N50 by 2.06%.

**Figure 2.**
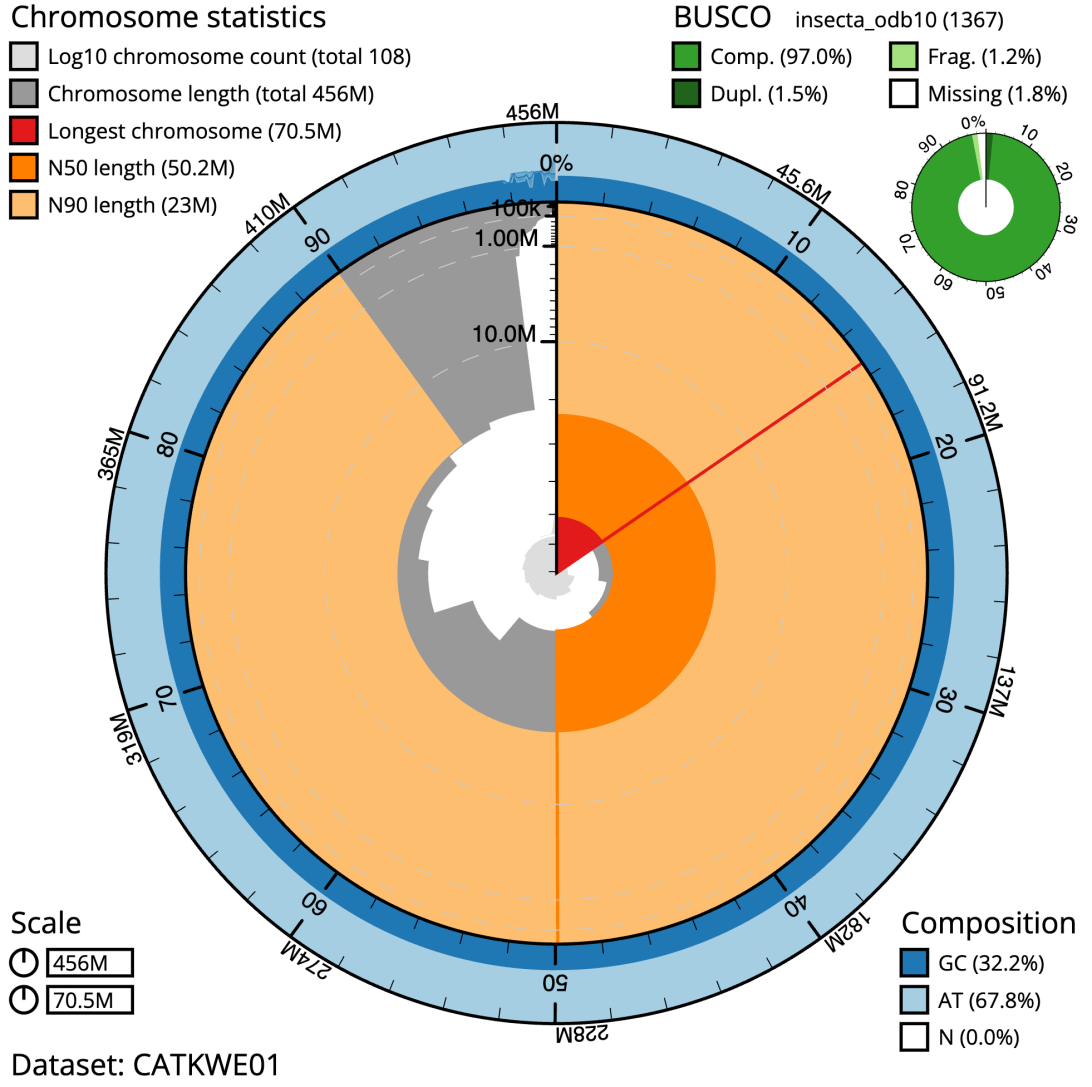
Genome assembly of *Siphlonurus alternatus*, ieSipAlte2.1: metrics. The BlobToolKit Snailplot shows N50 metrics and BUSCO gene completeness. The main plot is divided into 1,000 size-ordered bins around the circumference with each bin representing 0.1% of the 455,861,235 bp assembly. The distribution of scaffold lengths is shown in dark grey with the plot radius scaled to the longest scaffold present in the assembly (70,536,023 bp, shown in red). Orange and pale-orange arcs show the N50 and N90 scaffold lengths (50,231,148 and 22,999,722 bp), respectively. The pale grey spiral shows the cumulative scaffold count on a log scale with white scale lines showing successive orders of magnitude. The blue and pale-blue area around the outside of the plot shows the distribution of GC, AT and N percentages in the same bins as the inner plot. A summary of complete, fragmented, duplicated and missing BUSCO genes in the insecta_odb10 set is shown in the top right. An interactive version of this figure is available at https://blobtoolkit.genomehubs.org/view/Siphlonurus%20alternatus/dataset/CATKWE01/snail.

**Figure 3.**
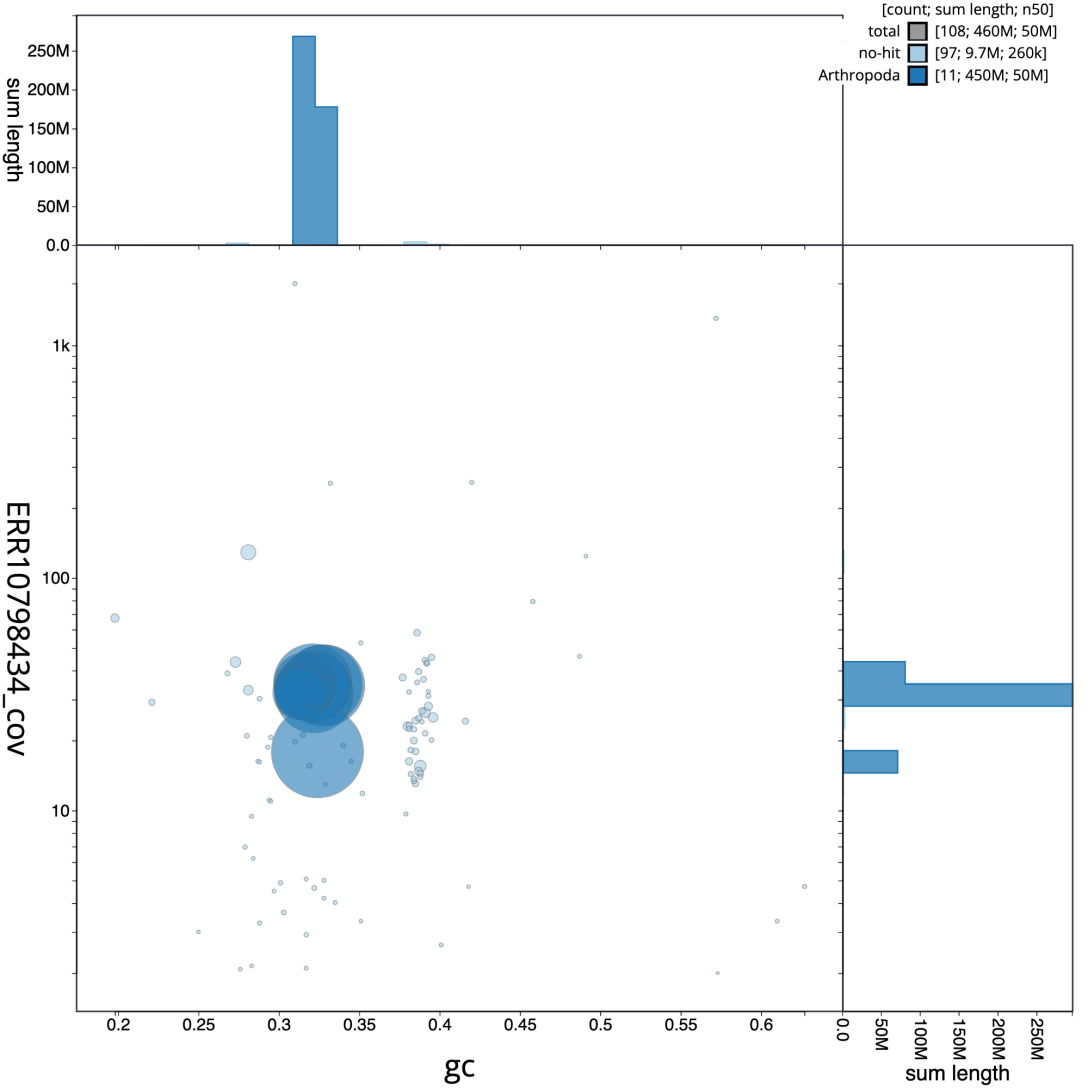
Genome assembly of *Siphlonurus alternatus*, ieSipAlte2.1: BlobToolKit GC-coverage plot. Scaffolds are coloured by phylum. Circles are sized in proportion to scaffold length. Histograms show the distribution of scaffold length sum along each axis. An interactive version of this figure is available at https://blobtoolkit.genomehubs.org/view/Siphlonurus%20alternatus/dataset/CATKWE01/blob.

**Figure 4.**
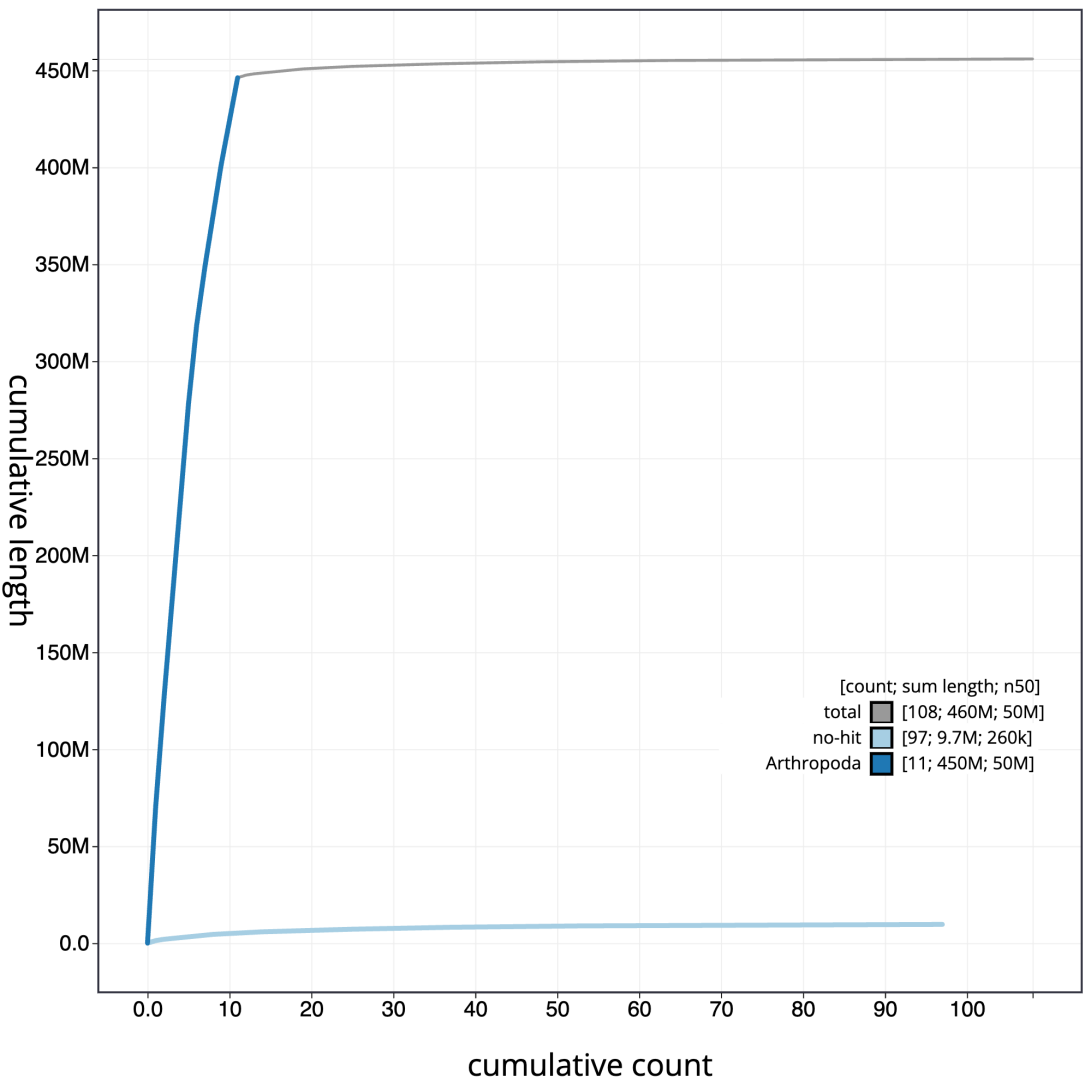
Genome assembly of *Siphlonurus alternatus*, ieSipAlte2.1: BlobToolKit cumulative sequence plot. The grey line shows cumulative length for all scaffolds. Coloured lines show cumulative lengths of scaffolds assigned to each phylum using the buscogenes taxrule. An interactive version of this figure is available at https://blobtoolkit.genomehubs.org/view/Siphlonurus%20alternatus/dataset/CATKWE01/cumulative.

**Figure 5.**
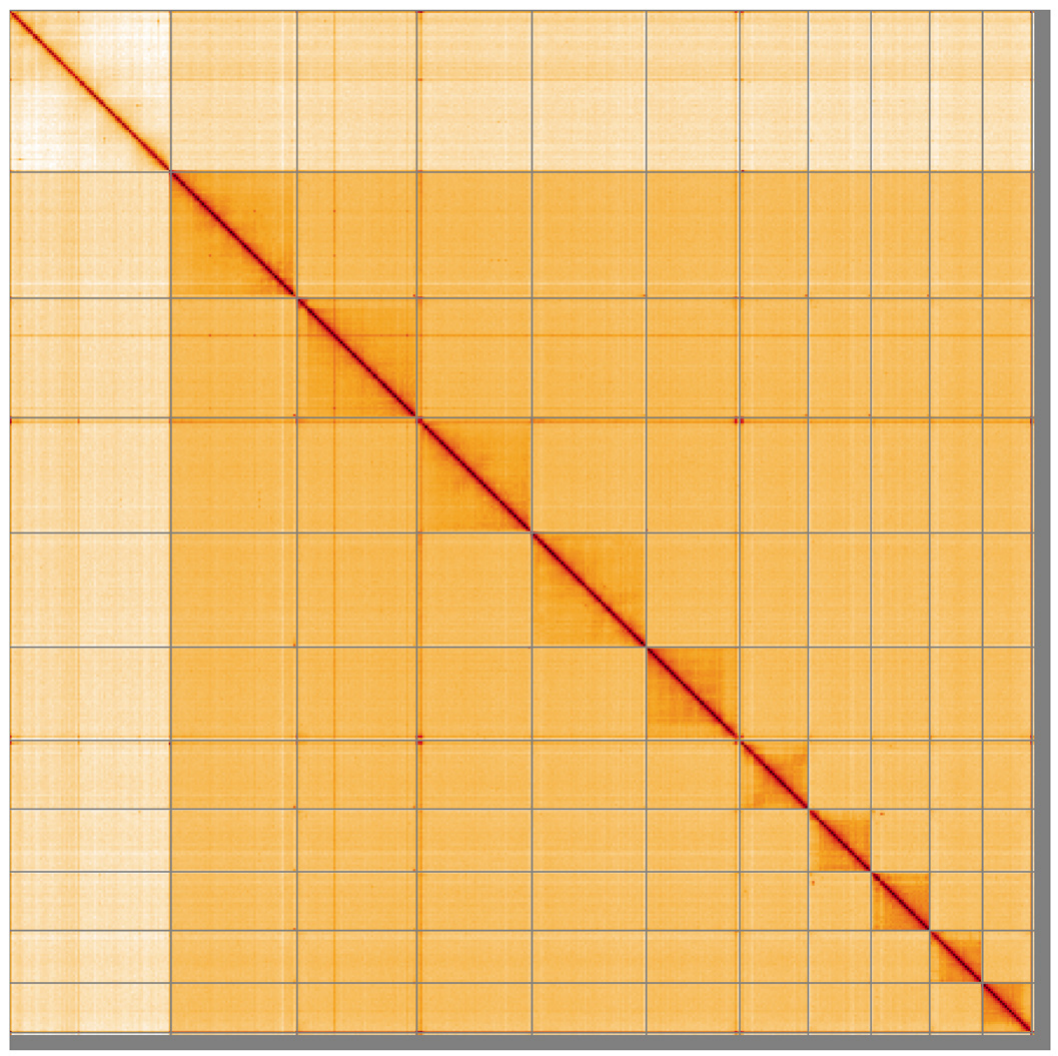
Genome assembly of *Siphlonurus alternatus*, ieSipAlte2.1: Hi-C contact map of the ieSipAlte2.1 assembly, visualised using HiGlass. Chromosomes are shown in order of size from left to right and top to bottom. An interactive version of this figure may be viewed at https://genome-note-higlass.tol.sanger.ac.uk/l/?d=MXAHm5u1RPiiYHsY51vQdQ.

The final assembly has a total length of 455.8 Mb in 107 sequence scaffolds with a scaffold N50 of 50.2 Mb ( Table 1). The snailplot in Figure 2 summarises the assembly statistics is shown in Figure 2, while the distribution of assembly scaffolds on GC proportion and coverage is shown in Figure 3. The cumulative assembly plot in Figure 4 shows curves for subsets of scaffolds assigned to different phyla. Most (97.87%) of the assembly sequence was assigned to 11 chromosomal-level scaffolds, representing 10 autosomes and the X sex chromosome. The X chromosome was identified by homology to *Sympetrum striolatum* (GCA_947579665.1) ( Crowley *et al.*, 2023) and coverage. The Y chromosome could not be uniquely identified in the unlocalised scaffolds. Chromosome-scale scaffolds confirmed by the Hi-C data are named in order of size ( Figure 5; Table 2). While not fully phased, the assembly deposited is of one haplotype. Contigs corresponding to the second haplotype have also been deposited. The mitochondrial genome was also assembled and can be found as a contig within the multifasta file of the genome submission.

**Table 1.** Genome data for *Siphlonurus alternatus*, ieSipAlte2.1.

Project accession data		
Assembly identifier	ieSipAlte2.1	
Species	*Siphlonurus alternatus*	
Specimen	ieSipAlte2	
NCBI taxonomy ID	248243	
BioProject	PRJEB59085	
BioSample ID	SAMEA110034127	
Isolate information	ieSipAlte2, male: whole organism (DNA sequencing and Hi-C scaffolding) ieSipAlte3: whole organism (RNA sequencing)	
Assembly metrics *		*Benchmark*
Consensus quality (QV)	54.3	*≥ 50*
*k*-mer completeness	99.99%	*≥ 95%*
BUSCO **	C:97.0%[S:95.5%,D:1.5%],F:1.2%,M:1.8%,n:1,367	*C ≥ 95%*
Percentage of assembly mapped to chromosomes	97.87%	*≥ 95%*
Sex chromosomes	X chromosome	*localised homologous pairs*
Organelles	Mitochondrial genome assembled	*complete single alleles*
Raw data accessions		
PacificBiosciences SEQUEL II	ERR10798434	
Hi-C Illumina	ERR10802458, ERR10802457	
PolyA RNA-Seq Illumina	ERR11837461	
Genome assembly		
Assembly accession	GCA_949825025.1	
*Accession of alternate haplotype*	GCA_947579545.1	
Span (Mb)	455.8	
Number of contigs	518	
Contig N50 length (Mb)	1.8	
Number of scaffolds	107	
Scaffold N50 length (Mb)	50.2	
Longest scaffold (Mb)	70.5	

* Assembly metric benchmarks are adapted from column VGP-2020 of “Table 1: Proposed standards and metrics for defining genome assembly quality” from ( Rhie *et al.*, 2021). ** BUSCO scores based on the insecta_odb10 BUSCO set using v5.3.2. C = complete [S = single copy, D = duplicated], F = fragmented, M = missing, n = number of orthologues in comparison. A full set of BUSCO scores is available at https://blobtoolkit.genomehubs.org/view/Siphlonurus%20alternatus/dataset/CATKWE01/busco.

**Table 2.** Chromosomal pseudomolecules in the genome assembly of *Siphlonurus alternatus*, ieSipAlte2.

INSDC accession	Chromosome	Length (Mb)	GC%
OX463778.1	1	55.02	33.0
OX463779.1	2	52.19	32.5
OX463780.1	3	50.23	32.0
OX463781.1	4	49.93	32.0
OX463782.1	5	40.73	32.0
OX463783.1	6	29.9	31.5
OX463784.1	7	27.39	31.5
OX463785.1	8	25.62	31.5
OX463786.1	9	23.0	31.5
OX463787.1	10	21.63	31.5
OX463788.1	X	70.54	32.5
OX463789.1	MT	0.02	31.0

The estimated Quality Value (QV) of the final assembly is 54.3 with *k*-mer completeness of 99.99%, and the assembly has a BUSCO v5.3.2 completeness of 97.0% (single = 95.5%, duplicated = 1.5%), using the insecta_odb10 reference set ( *n* = 1,367).

Metadata for specimens, spectra estimates, sequencing runs, contaminants and pre-curation assembly statistics can be found at https://links.tol.sanger.ac.uk/species/248243.

## Methods

### Sample acquisition and nucleic acid extraction

Specimens of *Siphlonurus alternatus* were collected from Drumpail Burn, Scotland, UK (latitude 54.92, longitude –4.77) on 2021-07-28. The specimens were collected and identified by Andrew Farr (independent researcher) and dry frozen at –80°C. One specimen (specimen ID NHMUK014543936, ToLID ieSipAlte2) was used for DNA sequencing and Hi-C data, and a second specimen (specimen ID NHMUK014543933, ToLID ieSipAlte3) was used for RNA sequencing.

DNA was extracted at the Tree of Life laboratory, Wellcome Sanger Institute (WSI). The ieSipAlte2 sample was weighed and dissected on dry ice with tissue set aside for Hi-C sequencing. Tissue from the whole organism was disrupted using a Nippi Powermasher fitted with a BioMasher pestle. High molecular weight (HMW) DNA was extracted using the Qiagen MagAttract HMW DNA extraction kit. HMW DNA was sheared into an average fragment size of 12–20 kb in a Megaruptor 3 system with speed setting 30. Sheared DNA was purified by solid-phase reversible immobilisation using AMPure PB beads with a 1.8X ratio of beads to sample to remove the shorter fragments and concentrate the DNA sample. The concentration of the sheared and purified DNA was assessed using a Nanodrop spectrophotometer and Qubit Fluorometer and Qubit dsDNA High Sensitivity Assay kit. Fragment size distribution was evaluated by running the sample on the FemtoPulse system.

RNA was extracted from whole organism tissue of ieSipAlte3 in the Tree of Life Laboratory at the WSI using TRIzol, according to the manufacturer’s instructions. RNA was then eluted in 50 μl RNAse-free water and its concentration assessed using a Nanodrop spectrophotometer and Qubit Fluorometer using the Qubit RNA Broad-Range (BR) Assay kit. Analysis of the integrity of the RNA was done using Agilent RNA 6000 Pico Kit and Eukaryotic Total RNA assay.

### Sequencing

Pacific Biosciences HiFi circular consensus DNA sequencing libraries were constructed according to the manufacturers’ instructions. Poly(A) RNA-Seq libraries were constructed using the NEB Ultra II RNA Library Prep kit. DNA and RNA sequencing was performed by the Scientific Operations core at the WSI on Pacific Biosciences SEQUEL II (HiFi) and Illumina NovaSeq 6000 (RNA-Seq) instruments. Hi-C data were also generated from remaining tissue of ieSipAlte2 using the Arima2 kit and sequenced on the Illumina NovaSeq 6000 instrument.

### Genome assembly, curation and evaluation

Assembly was carried out with Hifiasm ( Cheng *et al.*, 2021) and haplotypic duplication was identified and removed with purge_dups ( Guan *et al.*, 2020). The assembly was then scaffolded with Hi-C data ( Rao *et al.*, 2014) using YaHS ( Zhou *et al.*, 2023). The assembly was checked for contamination and corrected using the gEVAL system ( Chow *et al.*, 2016) as described previously ( Howe *et al.*, 2021). Manual curation was performed using gEVAL, HiGlass ( Kerpedjiev *et al.*, 2018) and Pretext ( Harry, 2022). The mitochondrial genome was assembled using MitoHiFi ( Uliano-Silva *et al.*, 2023), which runs MitoFinder ( Allio *et al.*, 2020) or MITOS ( Bernt *et al.*, 2013) and uses these annotations to select the final mitochondrial contig and to ensure the general quality of the sequence.

A Hi-C map for the final assembly was produced using bwa-mem2 ( Vasimuddin *et al.*, 2019) in the Cooler file format ( Abdennur & Mirny, 2020). To assess the assembly metrics, the *k*-mer completeness and QV consensus quality values were calculated in Merqury ( Rhie *et al.*, 2020). This work was done using Nextflow ( Di Tommaso *et al.*, 2017) DSL2 pipelines “sanger-tol/readmapping” ( Surana *et al.*, 2023a) and “sanger-tol/genomenote” ( Surana *et al.*, 2023b). The genome was analysed within the BlobToolKit environment ( Challis *et al.*, 2020) and BUSCO scores ( Manni *et al.*, 2021; Simão *et al.*, 2015) were calculated.

Table 3 contains a list of relevant software tool versions and sources.

**Table 3.** Software tools: versions and sources.

Software tool	Version	Source
BlobToolKit	4.0.7	https://github.com/blobtoolkit/blobtoolkit
BUSCO	5.3.2	https://gitlab.com/ezlab/busco
gEVAL	N/A	https://geval.org.uk/
Hifiasm	0.16.1-r375	https://github.com/chhylp123/hifiasm
HiGlass	1.11.6	https://github.com/higlass/higlass
Merqury	MerquryFK	https://github.com/thegenemyers/MERQURY.FK
MitoHiFi	2	https://github.com/marcelauliano/MitoHiFi
PretextView	0.2	https://github.com/wtsi-hpag/PretextView
purge_dups	1.2.3	https://github.com/dfguan/purge_dups
sanger-tol/genomenote	v1.0	https://github.com/sanger-tol/genomenote
sanger-tol/readmapping	1.1.0	https://github.com/sanger-tol/readmapping/tree/1.1.0
YaHS	yahs-1.1.91eebc2	https://github.com/c-zhou/yahs

### Wellcome Sanger Institute – Legal and Governance

The materials that have contributed to this genome note have been supplied by a Darwin Tree of Life Partner. The submission of materials by a Darwin Tree of Life Partner is subject to the **‘Darwin Tree of Life Project Sampling Code of Practice’**, which can be found in full on the Darwin Tree of Life website here. By agreeing with and signing up to the Sampling Code of Practice, the Darwin Tree of Life Partner agrees they will meet the legal and ethical requirements and standards set out within this document in respect of all samples acquired for, and supplied to, the Darwin Tree of Life Project. 

Further, the Wellcome Sanger Institute employs a process whereby due diligence is carried out proportionate to the nature of the materials themselves, and the circumstances under which they have been/are to be collected and provided for use. The purpose of this is to address and mitigate any potential legal and/or ethical implications of receipt and use of the materials as part of the research project, and to ensure that in doing so we align with best practice wherever possible. The overarching areas of consideration are:

•   Ethical review of provenance and sourcing of the material

•   Legality of collection, transfer and use (national and international)

Each transfer of samples is further undertaken according to a Research Collaboration Agreement or Material Transfer Agreement entered into by the Darwin Tree of Life Partner, Genome Research Limited (operating as the Wellcome Sanger Institute), and in some circumstances other Darwin Tree of Life collaborators.

## Data Availability

European Nucleotide Archive: *Siphlonurus alternatus*. Accession number PRJEB59085; https://identifiers.org/ena.embl/PRJEB59085. ( Wellcome Sanger Institute, 2023) The genome sequence is released openly for reuse. The *Siphlonurus alternatus* genome sequencing initiative is part of the Darwin Tree of Life (DToL) project. All raw sequence data and the assembly have been deposited in INSDC databases. The genome will be annotated using available RNA-Seq data and presented through the Ensembl pipeline at the European Bioinformatics Institute. Raw data and assembly accession identifiers are reported in Table 1.

## Author information

Members of the Natural History Museum Genome Acquisition Lab are listed here: https://doi.org/10.5281/zenodo.4790042.

Members of the Darwin Tree of Life Barcoding collective are listed here: https://doi.org/10.5281/zenodo.4893703.

Members of the Wellcome Sanger Institute Tree of Life programme are listed here: https://doi.org/10.5281/zenodo.4783585.

Members of Wellcome Sanger Institute Scientific Operations: DNA Pipelines collective are listed here: https://doi.org/10.5281/zenodo.4790455.

Members of the Tree of Life Core Informatics collective are listed here: https://doi.org/10.5281/zenodo.5013541.

Members of the Darwin Tree of Life Consortium are listed here: https://doi.org/10.5281/zenodo.4783558.
