# Supplementary material for: The genome sequence of the Northern Summer Mayfly, Siphlonurus alternatus (Say, 1824)
Source: Wellcome Open Res. 2023 Oct 23;8:488. [Version 1] doi: 10.12688/wellcomeopenres.20172.1 (PMC11069044; doi:10.12688/wellcomeopenres.20172.1)
Supplement: Peer review history

# Reviewer report: Reviewer response for version 1

**DOI:** 10.21956/wellcomeopenres.22335.r80525

**Published:** 2024-05-14

**Authors:** Kuppusamy Sivasankaran

**Affiliations:** 1 Entomology Research Institute, Loyola College, Chennai, Tamil Nadu, India

The genome of the Northern Summer Mayfly, *Siphlonurus alternatus* (Say, 1824) was sequenced and assembled using appropriate techniques. 

 Some minor comments

 In the background of the article the authors have given the genus name *Siphlonurus alternatus *full form continuously. First time the genus name can be given in full form then subsequently can be given in short form like *S. alternatus*.

 In the subheading “Genome sequence report” In the first line authors have mentioned “The genome was sequenced from one male *Siphlonurus alternatus*………. But authors have mentioned under the subheading “Sample acquisition and nucleic acid extraction” first line Specimens of *Siphlonurus alternatus *were collected from *………*

 Moreover authors have mentioned in the abstract “We present a genome assembly from an individual male *Siphlonurus alternatus………………..*

 There is a little confusion here because a specimen was used for DNA extraction and another specimen was used for RNA extraction.

 Page no six first paragraph third line “DNA and RNA sequencing was performed”. The sentence can be changed as DNA and RNA sequencing were performed.

 Above all, I confirm that the manuscript meets the necessary scientific standard and is suitable for indexing"

Are sufficient details of methods and materials provided to allow replication by others?

Yes

Is the rationale for creating the dataset(s) clearly described?

Yes

Are the datasets clearly presented in a useable and accessible format?

Yes

Are the protocols appropriate and is the work technically sound?

Yes

Reviewer Expertise:

Phylogenetic analysis of Noctuoidea moths

I confirm that I have read this submission and believe that I have an appropriate level of expertise to confirm that it is of an acceptable scientific standard.

# Reviewer report: Reviewer response for version 1

**DOI:** 10.21956/wellcomeopenres.22335.r80531

**Published:** 2024-05-14

**Authors:** Andrew J. Veale

**Affiliations:** 1 Manaaki Whenua Landcare Research, Lincoln, New Zealand

The assembly looks excellent and the data note is fine.  As per one of the previous reviewers, while RNA sequencing is mentioned in the methods it isn't mentioned in the results or discussion.  I assume it was used for annotation, but annotation was also not mentioned in the results or discussion?

 Was the second individual used for the RNA also male?  Also, what tissues were used for the transcriptomes?

 Mentioning that this was a male (I believe?), and that mayflies have a male XY female XX sex determination may be useful for those that do not know this.

 Other than that it is fine.

Are sufficient details of methods and materials provided to allow replication by others?

Yes

Is the rationale for creating the dataset(s) clearly described?

Yes

Are the datasets clearly presented in a useable and accessible format?

Yes

Are the protocols appropriate and is the work technically sound?

Yes

Reviewer Expertise:

Wildlife ecology, genomics, conservation.

I confirm that I have read this submission and believe that I have an appropriate level of expertise to confirm that it is of an acceptable scientific standard, however I have significant reservations, as outlined above.

# Reviewer report: Reviewer response for version 1

**DOI:** 10.21956/wellcomeopenres.22335.r80523

**Published:** 2024-05-03

**Authors:** Jerome H. L Hui

**Affiliations:** 1 The Chinese University of Hong Kong, Hong Kong, Hong Kong

Farr and colleagues report the genome sequence of mayfly Siphlonurus alternatus (Say, 1824). Early record of this species in Britain could date back to 1913 (Macadam 2019, The Glasgow Naturalist). Molecular data of this species are scarce prior to this report, and are mainly confined to COI sequences deposited to the NCBI database. Therefore, this new genome resource will be useful for further studies, ranging from revealing their population structures, ecology, understanding their evolution with other insects, as well as studying the impact of climate change on them.

 This genome resource is excellent from the summary statistics, with high BUSCO numbers, high sequence continuity, and majority of sequences contained on 11 pseudochromosomes (plus mitochondrion). All in all, this is another valuable contribution carried out by the Darwin Tree of Life Consortium.

Are sufficient details of methods and materials provided to allow replication by others?

Yes

Is the rationale for creating the dataset(s) clearly described?

Yes

Are the datasets clearly presented in a useable and accessible format?

Yes

Are the protocols appropriate and is the work technically sound?

Yes

Reviewer Expertise:

Genomics, evolution, invertebrates

I confirm that I have read this submission and believe that I have an appropriate level of expertise to confirm that it is of an acceptable scientific standard.

# Reviewer report: Reviewer response for version 1

**DOI:** 10.21956/wellcomeopenres.22335.r80530

**Published:** 2024-05-03

**Authors:** Diego De Panis

**Affiliations:** 1 Leibniz Institute for Zoo and Wildlife Research, Berlin, Germany

The article shows the high-quality reference genome of an insect.

 The asm procedure is standard dtol. Many of the points raised by rev1 are available in the link provided in the last sentence of the Genome sequence report section: "Metadata for specimens, spectra estimates, sequencing runs, contaminants and pre-curation assembly statistics can be found at https://links.tol.sanger.ac.uk/species/248243". No need to add those in the article.

 The results are above the recommended EBP metrics, and the HiC contact map looks clean after curation.

 I think the information available is appropriate for this type of article, but in the same direction as commented by rev1, the article is missing a sentence or two expanding on the objectives and applications.

 Finally, the article lacks information about the use of the RNA-Seq dataset generated from the specimen ieSipAlte3 (is it a male, like ieSipAlte2?).

 The assembly looks very good, the article is appropriate for this data.

Are sufficient details of methods and materials provided to allow replication by others?

Yes

Is the rationale for creating the dataset(s) clearly described?

Partly

Are the datasets clearly presented in a useable and accessible format?

Yes

Are the protocols appropriate and is the work technically sound?

Yes

Reviewer Expertise:

Genomics

I confirm that I have read this submission and believe that I have an appropriate level of expertise to confirm that it is of an acceptable scientific standard, however I have significant reservations, as outlined above.

# Reviewer report: Reviewer response for version 1

**DOI:** 10.21956/wellcomeopenres.22335.r76669

**Published:** 2024-03-29

**Authors:** Marta Coronado-Zamora

**Affiliations:** 1 Institute of Evolutionary Biology, CSIC, UPF, Barcelona, Spain

This data note describes a high quality chromosomal assembly of the Northern Summer Mayfly, *Siphlonurus alternatus*, using the most advanced sequencing and assembly methods. The genome data are available at NCBI BioProject, and data is accessible (as checked for some of the chromosomes). 

***Comments***:

 I miss in the background more description on the rationale for generating this assembly. The only reference to that is the last sentence " *The genome sequence for Siphlonurus alternatus will aid in understanding the biology, physiology and ecology of the species.*". If I'm not mistaken, this is the first genome available for this species, this could be mentioned too. 

 RNA-seq is also generated for this species, however, this is not mentioned in the Results section. It would be helpful to describe how the RNA-seq was used or will be used (as mentioned in the Data availability will be used for gene annotation, but this could be mentioned earlier).

***Small comments and typos***:

 - First sentence of the Background: "northern United States": Northern should be capitalized.

 - *Siphlonurus alternatus* could be shortened to *S. alternatus *after the first mention in each section.

 - In the first sentence of the Genome sequence report, the year of collection could be added (after e.g. the coordinates of sampling).

 - Provide the N50 statistics after the manual assembly curation in "A total of 53-fold coverage in ... was generated (N50=)".

 - Fix the sentence "The snailplot in Figure 2 summarises the assembly statistics is shown in Figure 2".

 - Figure 2: Why it reads "Dataset: CATKWE01" in the bottom left?

 - Figures 2-4 in the PDF version are too big and take a lot of space of the page.

 - "bwa-mem" is missing from Table 3

 - For reproducibility of the assembly, I miss the options/parameters used in the programs and tools. This could be specified in Table 3. I also suggest sharing scripts used through e.g. GitHub.

Are sufficient details of methods and materials provided to allow replication by others?

Yes

Is the rationale for creating the dataset(s) clearly described?

Partly

Are the datasets clearly presented in a useable and accessible format?

Yes

Are the protocols appropriate and is the work technically sound?

Yes

Reviewer Expertise:

bioinformatics, population genetics, epigenetics, transposable elements, adaptation

I confirm that I have read this submission and believe that I have an appropriate level of expertise to confirm that it is of an acceptable scientific standard, however I have significant reservations, as outlined above.
